# Regional and Age-Related Variations in Blood Calcium Levels among Patients with *Plasmodium falciparum* and *P. vivax* malaria: A Systematic Review and Meta-Analysis

**DOI:** 10.3390/nu15214522

**Published:** 2023-10-25

**Authors:** Kwuntida Uthaisar Kotepui, Aongart Mahittikorn, Polrat Wilairatana, Frederick Ramirez Masangkay, Manas Kotepui

**Affiliations:** 1 Medical Technology, School of Allied Health Sciences, Walailak University, Thasala, Nakhon Si Thammarat 80160, Thailand; 2 Department of Protozoology, Faculty of Tropical Medicine, Mahidol University, Bangkok 10400, Thailand; 3 Department of Clinical Tropical Medicine, Faculty of Tropical Medicine, Mahidol University, Bangkok 10400, Thailand; 4 Department of Medical Technology, Faculty of Pharmacy, University of Santo Tomas, Manila 1008, Philippines

**Keywords:** malaria, calcium, micronutrients, *Plasmodium*, meta-analysis

## Abstract

Despite several studies examining the relationship between calcium levels and malaria, inconsistencies and varied results remain in the literature. This study aimed to synthesize the evidence on the association between blood calcium levels and malaria severity. A systematic literature search was conducted in the Embase, Scopus, PubMed, Ovid, and Google Scholar databases. The studies that investigated calcium levels in participants with malaria were reviewed and included for synthesis. The quality of included studies was assessed based on a standardized checklist by the Joanna Briggs Institute (JBI) critical appraisal checklists. The thematic synthesis had been used for qualitative synthesis. For the quantitative synthesis, the meta-analysis was performed to estimate the pooled effect sizes for differences in calcium levels between groups of participants using a random effect model using Hedge’s g as a measure of effect size. Out of the 4574 identified records, 14 studies were reviewed. The thematic synthesis across these studies noted a consistent theme: reduced calcium levels in malaria patients compared to uninfected controls. However, the meta-analysis encompassing three specific analyses—comparing calcium levels between malaria patients and controls, severe and non-severe malaria cases, and fatal cases versus survivors—showed no significant difference in calcium levels. The statistics were as follows: (1) *p* = 0.15, Hedge’s g: −1.00, 95% CI: −2.37–0.38, *I*^2^: 98.97, 9 studies; (2) *p* = 0.35, Hedge’s g: −0.33, 95% CI: −1.02–0.36, *I*^2^: 81.61, 3 studies; and (3) *p* = 0.71, Hedge’s g: −0.14, 95% CI: −0.91–0.62, *I*^2^: 87.05, 3 studies. Subgroup analyses indicated that regional disparities, especially between Africa and Asia, and participant age groups may influence these outcomes. While a trend of decreased calcium levels in malaria patients was observed, the meta-analytical results suggest regional and age-related variations. Further investigations should emphasize these differences to better guide clinical management, prognostic applications, and the crafting of policies concerning malaria’s metabolic effects.

## 1. Introduction

Malaria remains a significant global health challenge, with 247 million cases and 625,000 deaths in 2021, predominantly in sub-Saharan Africa [1]. This disease is caused by protozoan parasites of the genus *Plasmodium* and is transmitted through the bite of an infected female *Anopheles* mosquito [2]. With five main species known to infect humans—*P. falciparum*, *P. vivax*, *P. ovale*, *P. malariae*, and *P. knowlesi*—each presents varying severity and clinical manifestations [1]. Notably, *P. falciparum* is the leading cause of severe malaria and related deaths. However, other species can occasionally cause severe forms of the disease [3,4]. Understanding the intricate pathogenesis of malaria, from the initial mosquito bite to the systemic manifestations in the human host, is vital for developing innovative strategies to counteract its debilitating effects and reduce its global impact.

Calcium is a vital mineral with diverse physiological roles, from contributing to bone health to facilitating intracellular signaling, blood clotting, muscle contraction, and nerve function [5,6,7]. Its homeostasis is meticulously regulated by various hormonal pathways involving the parathyroid hormone, vitamin D, and calcitonin, ensuring consistent serum calcium levels critical for the body’s overall function [8,9,10]. The pathogenesis of intracellular pathogens has been linked to their ability to manipulate the host cell’s calcium signaling to enhance their survival and replication [11,12]. Moreover, these pathogens can induce changes in intracellular calcium levels, affecting host cell apoptosis, cytokine production, and other immune responses [13]. Significantly, disruptions in calcium homeostasis have been documented in several infectious diseases, including toxoplasmosis [14], tuberculous peritonitis [15,16], coronavirus disease (COVID-19) [17,18,19], and malaria [20,21], underscoring calcium’s potential role in the pathophysiology of these diseases including malaria.

While several studies have explored the relationship between calcium levels and malaria, inconsistencies and varied results persist in the literature [20,21,22]. Therefore, alterations in blood calcium levels were hypothesized to be associated with the severity of malaria in patients. A systematic review and meta-analysis can provide a comprehensive and clearer understanding by pooling these disparate findings. This consolidated approach not only enhances our insight into the pathophysiology of malaria and its impact on calcium levels but also pinpoints potential therapeutic targets. Furthermore, a meta-analysis can amplify the precision of estimates by aggregating data from multiple studies, thus revealing associations that might be overlooked in individual studies due to limited statistical power. The present study aimed to synthesize evidence regarding the association between blood calcium levels and the severity of malaria in patients.

## 2. Materials and Methods

### 2.1. Protocol and Registration

The systematic review has been registered with PROSPERO under the code CRD42023464711. A systematic literature review was conducted following the Preferred Reporting Items for Systematic Reviews and Meta-Analyses (PRISMA) guidelines [23] to explore the association between blood calcium levels and malaria.

### 2.2. Outcomes

The outcomes of the study were the differences in blood calcium levels between: (i) patients with malaria and uninfected individuals; (ii) patients with severe malaria and those with non-severe malaria; and (iii) fatal malaria cases and survivors.

### 2.3. Systematic Review Question

The Population, Exposure, Comparator, Outcome (PECO) framework was used to develop the systematic review question [24]. ‘P’ represented participants in malaria-endemic areas; ‘E’ signified *Plasmodium* infection, severe, or fatal malaria; ‘C’ denoted uninfected participants, those with less severe malaria, or survivors; and ‘O’ corresponded to blood calcium levels.

### 2.4. Search Strategy

A systematic literature search was conducted across four primary databases: Embase, Scopus, PubMed, and Ovid. The search strategy, which included the terms ‘calcium’ AND ‘malaria’ OR ‘Plasmodium’ OR ‘Plasmodium Infection’ OR ‘Remittent Fever’ OR ‘Marsh Fever’ OR ‘Paludism’, was employed to identify studies investigating the relationship between calcium levels and malaria. The search terms and their synonyms were identified using Medical Subject Headings (MeSH). The search strategy varied slightly across different databases (see Table S1). Studies were sought without any language, geographical, or publication year restrictions. To ensure the comprehensiveness of the search, Google Scholar was reviewed, and the reference lists of included articles were searched. The searches spanned from 6 May 2023 to 11 September 2023.

### 2.5. Inclusion and Exclusion Criteria

Studies were eligible for inclusion if they (i) investigated calcium levels in participants with malaria; (ii) compared calcium levels between patients with malaria and uninfected individuals or investigated the relationship between calcium levels and severity or type of malaria; (iii) were cross-sectional, case-control, cohort, or clinical trials. Reports were excluded if they were in vitro, animal, proteomic, in silico/docking, computational, genomic studies, reviews, case reports, lacked relevant data or if the data were impossible to extract.

### 2.6. Study Selection and Data Extraction

Study selection began with the removal of duplicates, and then titles and abstracts of articles were screened. Full-text articles of relevant articles were assessed, leading to the exclusion of non-relevant articles for specific reasons. Extracted information encompassed study characteristics like publication year, design, geographical location, *Plasmodium* species studied, participant demographics, symptomatology, severity of malaria, methods for *Plasmodium* identification, and the blood sample used for calcium measurements. Two independent reviewers selected studies based on eligibility criteria and extracted data from the included studies, with any disagreements being resolved through discussion.

### 2.7. Quality Assessment of Included Studies

The quality of included studies was assessed based on a standardized checklist by the Joanna Briggs Institute (JBI) critical appraisal checklists [25]. For cross-sectional studies, this checklist evaluated clarity in methodology, the handling of confounding factors, and statistical analysis, among other criteria. For case-control studies, criteria such as group comparability, case identification, exposure measurement, outcome assessment, and handling of confounding factors were considered. Cohort studies were evaluated for similarities between groups, consistent exposure measurement, addressing of confounding factors, follow-up time, and strategies to handle incomplete follow-ups. Clinical trials were assessed based on criteria like randomization, baseline group similarity, outcomes measurement, blinding, and appropriate statistical analysis. Two independent reviewers assessed studies based on specific checklists, with any disagreements being resolved through discussion.

### 2.8. Data Syntheses

The thematic synthesis [26] had been used for qualitative synthesis based on geographical location, *Plasmodium* species, age groups, and disease severity. For the quantitative synthesis, calcium levels and their variability measures (mean/median, standard deviation/range) were extracted. When these values were not directly provided, they were calculated or estimated using available data as described previously [27]. For statistical analysis, meta-analyses were performed to estimate the pooled effect sizes for differences in calcium levels between groups using random effect model [28]. Hedge’s g was used as a measure of effect size for continuous outcomes. The primary outcome was to estimate the difference in calcium levels between patients with malaria and uninfected controls. The secondary outcome was to assess the difference in calcium levels between patients with severe malaria and those without. The tertiary outcome was to assess the difference in calcium levels between fatal cases and survivors. For each meta-analysis, a forest plot was generated to visually represent the effect size and associated confidence intervals. Heterogeneity among studies was assessed using the *I*^2^ statistic, which describes the proportion of the total variation across studies that is due to heterogeneity rather than chance [29]. The heterogeneity among the studies was evaluated using the *I*^2^ statistic, where values exceeding 50% suggest significant heterogeneity [29]. The meta-regression was applied to identify potential sources of bias (study-level covariates) that might impact the results and to provide a more nuanced understanding of the relationships between blood calcium levels and malaria severity. Several potential sources of bias including publication years, study design, study area, age group, *Plasmodium* species, symptoms, methods for *Plasmodium* identification, and blood samples for calcium measurement were assessed in the meta-regression analyses. Subgroup analyses were performed to explore potential effect modifiers. A leave-one-out meta-analysis approach was employed to understand the impact of each individual study on the overall pooled effect estimate [30]. This analysis aimed to test the robustness of the meta-analysis findings by examining how the pooled effect estimate would change when each study was systematically excluded. All analyses were conducted using appropriate Stata v17.0 software (StataCorp, College Station, TX, USA), with *p*-values less than 0.05 considered statistically significant.

## 3. Results

### 3.1. Search Results

**Figure 1:**
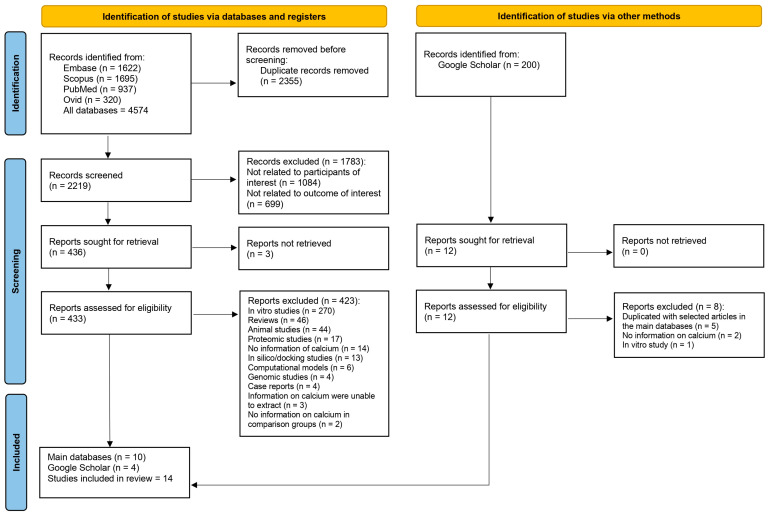
Study flow diagram.

A total of 4574 records from databases were identified: 1622 from Embase, 1695 from Scopus, 937 from PubMed, and 320 from Ovid. Before screening, 2355 duplicate records were removed, leaving 2219 records for screening. Upon screening, 1783 records were excluded for specific reasons: 1084 were not related to the participants of interest, and 699 were not related to the outcome of interest. This led to 436 reports being sought for retrieval. Out of these, 3 reports could not be retrieved, resulting in 433 reports assessed for eligibility. Among the assessed reports, 423 were excluded for the following reasons: 270 were in vitro studies, 46 were reviews, 44 were animal studies, 17 were proteomic studies, 14 had no information on calcium, 13 were in silico/docking studies, 6 were computational models, 4 were genomic studies, 4 were case reports, 3 had data on calcium that were impossible to extract, and 2 lacked calcium data in comparison groups. Besides the 10 reports from the main databases that met the eligibility criteria, 4 reports were found from Google Scholar. In total, 14 studies were included in the review [20,21,22,31,32,33,34,35,36,37,38,39,40,41] (Figure 1).

### 3.2. Characteristics of Included Studies

Among the 14 studies included in the review, a breakdown by publication year revealed that 28.57% were published before 2000, 37.71% between 2000 and 2009, 28.54% between 2010 and 2019, and 7.14% from 2020 to 2023. According to the study designs, the majority (57.14%) were cross-sectional, 28.57% were case-control studies, and both the cohort studies and clinical trials each made up 7.14%. Geographically, the studies were equally divided between Asia and Africa, representing 50% each. In Asia, the breakdown was as follows: India (21.43%), Vietnam (14.29%), Thailand (7.14%), and China (7.14%). For the African region, Nigeria was the most represented at 21.43%, followed by Cameroon at 14.29%, with Sudan and Kenya each contributing 7.14%. According to *Plasmodium* species, a significant majority (71.43%) focused on *P. falciparum*, 14.29% on mixed infections of *P. falciparum* and *P. vivax*, 7.14% on both *P. falciparum* and *P. vivax*, and 7.14% did not specify the species. The participant demographics were predominantly adults at 50%, children made up 35.71%, and a combination of children and adults represented 14.29%. Symptomatic cases constituted 78.57% of the studies, while the severity of the malaria was distributed among severe malaria (21.43%), a combination of severe and non-severe malaria (42.86%), non-severe cases such as uncomplicated, mild, or asymptomatic malaria (7.14%), and 28.57% did not specify the severity. The methods of *Plasmodium* identification primarily utilized the microscopic method at 64.29%, with a combination of microscopic and RDT in 14.29%, and microscopic combined with quantitative buffy coat in 7.14%. The blood samples used for calcium were mainly serum (64.29%) and plasma (35.71%) (Table 1 and Table S2).

### 3.3. Quality of Included Studies

For eight cross-sectional studies, one study had certain ambiguities regarding confounding factors but was generally clear in other areas [20]. Two studies fully adhered to all the checklist criteria [33,34]. One study also met all the standards set by the checklist [38]. Two studies [21,39] provided clear methodologies but overlooked confounding factors. Another study was ambiguous in various criteria, particularly in addressing confounding factors and statistical analysis [40]. One study clearly addressed most criteria but was lacking in terms of its handling of confounding factors [22]. For four case-control studies, one study had ambiguities in group comparability and in the matching of cases and controls but was consistent in identifying cases, measuring exposure, and assessing outcomes [31]. One study was thorough in most criteria, except in addressing confounding factors [32]. One study exhibited a comprehensive adherence to all the checklist criteria [36]. Another study was clear in most aspects but had uncertainties about confounding factors [41]. A cohort study demonstrated a strong adherence to most of the criteria. The study ensured that groups were similar, exposures were consistently measured, and confounding factors were both identified and addressed [35]. However, there were ambiguities related to follow-up time, completeness of follow-up, and strategies to handle incomplete follow-ups, though it was still deemed fit for inclusion. A clinical trial showcased clear adherence to true randomization, baseline group similarity, and outcomes measurement [37]. While some aspects like blinding and follow-up completeness were unclear, the study did utilize appropriate statistical analysis and the trial design was deemed suitable (Table S2).

### 3.4. Thematic Synthesis

For geographical patterns, the studies in Africa, such as in Sudan [20], Nigeria [31,32], Cameroon [36], and Kenya [37], predominantly observed *P. falciparum* as the causative *Plasmodium* species in malaria. The studies in Asia, such as in Thailand [34], China [33], Vietnam [35,41], and India [22,39,40], observed both *P. falciparum* and *P. vivax* infections, with some mixed infections. For the difference in calcium levels between patients with malaria and uninfected controls, a notable theme across many studies, irrespective of continent, was the significant decrease in calcium levels in malaria patients compared to uninfected controls. This observation was consistent in both children and adults in a group of studies [20,31,32,41]. However, there were exceptions. For instance, two studies [34,35] found scenarios where calcium levels did not differ significantly based on the severity of the disease or survival outcome.

For the disease severity and calcium levels, the study from Nigeria showed a correlation between disease severity and calcium levels, with the latter decreasing with increasing severity from mild to severe malaria [21]. Contrarily, the study from Cameroon did not observe a significant difference in calcium levels between varying severities of the disease [38]. Davis et al. (1991) reported increased calcium levels in patients who succumbed to the disease compared to those who survived, offering a unique perspective amidst other studies that did not observe such distinctions [34] (Table 2). While a generalized decrease in calcium levels among patients with malaria was a consistent observation, specifics fluctuate based on factors like disease severity, demographic profiles, and *Plasmodium* species.

### 3.5. Meta-Analysis of the Difference in Calcium Levels in Relation to Malaria

**Figure 2:**
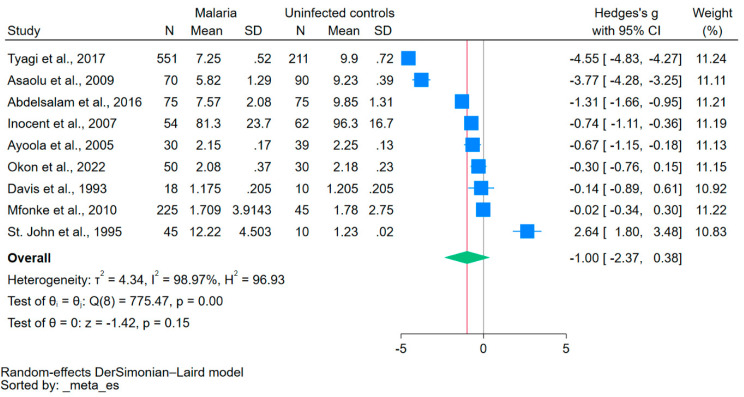
The forest plot showed no significant difference in calcium levels between patients with malaria and uninfected controls (*p* = 0.15). Blue square, effect estimate; green diamond, pooled effect estimate; gray vertical line, no effect line; red vertical line, pooled effect estimate; N, number of participants; mean, mean calcium levels; SD, standard deviation of calcium levels; CI, confidence interval. References [20,21,22,31,32,33,36,38,41].

Using data from nine studies that reported mean/median and standard deviation/range of calcium levels [20,21,22,31,32,33,36,38,41], the difference in calcium levels between patients with malaria and uninfected controls was estimated. The results showed no significant difference in calcium levels between the two groups (*p* = 0.15, Hedge’s g: −1.00, 95% CI: −2.37–0.38, *I*^2^: 98.97, 9 studies, Figure 2).

Given the high heterogeneity of the effect estimates across the included studies, a meta-regression was performed using factors such as publication years, study design, continent, age group, *Plasmodium* species, clinical status, diagnostic method for *Plasmodium* species, and blood samples for calcium measurements as covariates. The results indicated that both age group and *Plasmodium* species influenced the pooled effect estimate, suggesting these factors play a role in varying calcium levels in patients with malaria (Table S4).

Subgroup analyses of calcium levels in patients with malaria, compared to uninfected controls, showed varied results across categories. For publication years, there was no significant difference in calcium levels across all year ranges (*p* > 0.05). A similar non-significance was observed across different study designs (*p* > 0.05). Geographically, studies from Africa displayed a significant pooled effect estimate (*p* = 0.02), whereas studies from Asia did not show any significant difference (*p* = 0.76). Regarding age groups, a significant pooled effect was observed in children (*p* = 0.02), but not in adults (*p* = 0.55). Finally, there was no significant difference in calcium levels among studies using various methods for *Plasmodium* identification or different blood samples for calcium measurement (*p* > 0.05, Table 3).

### 3.6. Meta-Analysis of the Difference in Calcium Levels in Relation to Severe Malaria

**Figure 3:**
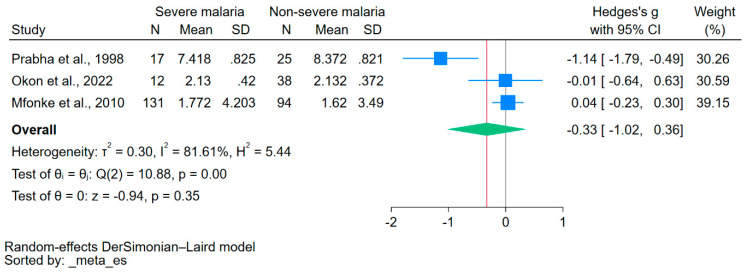
The forest plot showed no significant difference in calcium levels between patients with severe malaria and those without severe malaria (*p* = 0.35). Blue square, effect estimate; green diamond, pooled effect estimate; gray vertical line, no effect line; red vertical line, pooled effect estimate; N, number of participants; mean, mean calcium levels; SD, standard deviation of calcium levels; CI, confidence interval. References [21,38,40].

Using data from three studies that reported on the mean/median and standard deviation/range of calcium levels [21,38,40], the difference in calcium levels between patients with severe malaria and those without severe malaria was estimated. The results showed no significant difference in calcium levels between the two groups (*p* = 0.35, Hedge’s g: −0.33, 95% CI: −1.02–0.36, *I*^2^: 81.61, 3 studies, Figure 3).

### 3.7. Meta-Analysis of the Difference in Calcium Levels in Relation to Fatal Malaria

**Figure 4:**
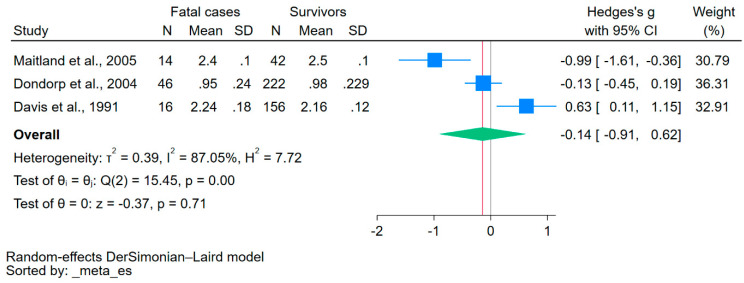
The forest plot showed no significant difference in calcium levels between fatal cases and survivors (*p* = 0.71). Blue square, effect estimate; green diamond, pooled effect estimate; gray vertical line, no effect line; red vertical line, pooled effect estimate; N, number of participants; mean, mean calcium levels; SD, standard deviation of calcium levels; CI, confidence interval. References [34,35,37].

Using data from three studies that reported on the mean/median and standard deviation/range of calcium levels [34,35,37], the difference in calcium levels between fatal cases and survivors was estimated. The results showed no significant difference in calcium levels between the two groups (*p* = 0.71, Hedge’s g: −0.14, 95% CI: −0.91–0.62, *I*^2^: 87.05, 3 studies, Figure 4).

### 3.8. Sensitivity Analysis

**Figure 5:**
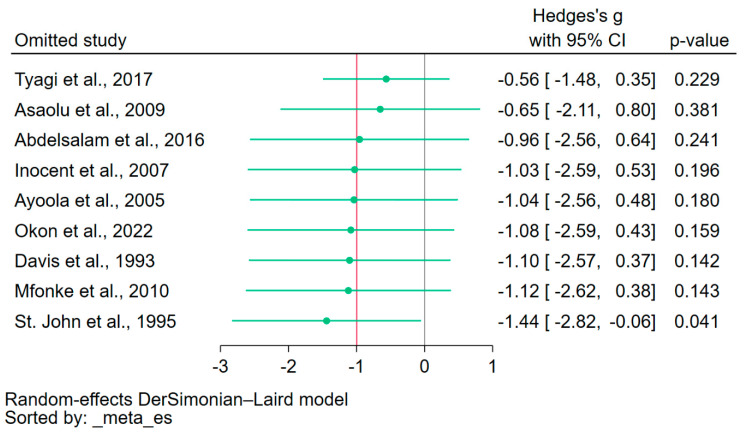
Leave-one-out sensitivity analysis. The figure illustrated the results of the leave-one-out meta-analysis, where each study was sequentially excluded to assess its individual influence on the overall pooled effect estimate. The observed fluctuation in the effect estimates when each study was excluded highlighted the instability of the meta-analysis. Green dot, pooled effect estimate; green horizontal line, confidence interval. Gray vertical line, no effect line; red vertical line, pooled effect estimate; References [20,21,22,31,32,33,36,38,41].

The leave-one-out meta-analysis approach was employed to assess the influence of each individual study on the overall pooled effect estimate. By systematically excluding one study at a time and recalculating the pooled estimate, the results indicated variability in the pooled effect estimate, revealing the sensitivity of the meta-analysis to the exclusion of individual studies (Figure 5). The leave-one-out meta-analysis, which assessed the difference in calcium levels between patients with severe malaria and those without, as well as between fatal cases and survivors, was not conducted because only three studies were included in the meta-analysis.

## 4. Discussion

The systematic review and meta-analysis aimed to explore the relationship between calcium levels and malaria, a relationship which has hitherto been underexplored in the literature. The review exposed distinct patterns, intricacies, and consistencies surrounding this relationship. A consistent theme emerged across studies from different continents; malaria patients, both children and adults, generally displayed decreased calcium levels compared to uninfected controls. This decrease may hold potential implications for patient management, although its clinical significance remains to be elucidated further. However, the uniformity in this observation was punctuated by a couple of studies which did not find significant differences based on disease severity or survival outcomes, emphasizing that factors beyond geographical location might influence this association. Interestingly, while individual studies showcased associations between disease severity and calcium levels, the meta-analysis results did not establish significant differences in calcium levels between patients with malaria and uninfected controls, or even between different severities of malaria or survival outcomes. The high heterogeneity observed across the studies could account for these findings. Factors such as age group and *Plasmodium* species appeared to significantly influence the pooled effect estimates, underscoring their potential role in dictating calcium dynamics in the context of malaria.

The decline in calcium levels may be linked to the clinical manifestations of malaria, which impact neuromuscular excitability, nerve transmission, and muscle contraction [21]. The renal dysfunction caused by *Plasmodium* infection can lead to an increase in the urinary excretion of minerals, especially calcium [42]. A significant reduction in calcium concentration in malaria patients might be associated with the cytoadherence of parasitized erythrocytes in the glomerular capillaries, which could intensify calcium excretion through urine [31]. Interestingly, one study reported that, while decreased calcium was rare, heightened serum calcium levels were commonly observed upon admission, affecting 31% of patients [37]. Moreover, *Plasmodium* trophozoites are known to sequester calcium within their internal compartments for metabolic activities, potentially contributing to the observed hypocalcemia in patients with malaria [43]. This observation aligns with findings where parasitized erythrocytes contained elevated intracellular calcium levels [44]. Another study postulated that the lysis of erythrocytes could prompt a surge in calcium levels due to the intracellular release of this mineral [37]. Supporting this, there was evidence of an inverse relationship between decreased hemoglobin levels and increased calcium levels in adults with severe malaria [34]. Additionally, a notable decrease in calcium can also arise from diminished vitamin D levels during infections, subsequently leading to reduced calcium levels [42]. In relation to magnesium, its concentration was found to be significantly lower in cases of high parasitemia compared to moderate and low levels [20,40], suggesting that reduced magnesium might be linked to an elevated risk of complications and severe disease manifestations. Recovery from malaria has been associated with the normalization of blood calcium levels [40], indicating its potential role as a prognostic biomarker for the disease.

Subgroup analyses further refined the understanding of results obtained through the meta-analysis. While studies across different publication years and designs did not show significant variances in calcium levels, geographical distinction emerged as a pivotal factor. African studies revealed a significant reduction in calcium levels in patients with malaria, implying a more pronounced relationship between malaria and calcium levels in this region compared to Asia. Several factors could contribute to the more pronounced reduction in calcium levels in patients with malaria from one region compared to another, such as Africa versus Asia. The first factor was *P. falciparum*, which might be more prevalent in Africa than in Asia, leading to distinct clinical outcomes and influences on calcium metabolism. The second factor was the coexisting nutritional deficiencies in Africa, such as differences in the dietary intake of calcium or Vitamin D, could make certain populations more susceptible to hypocalcemia when infected with malaria [45,46,47]. Another factor was the presence of other concurrent infections, which might be more common in Africa, which could also influence calcium metabolism and levels [48,49,50]. It is important to note that these are potential explanations, and the actual reasons, would require rigorous scientific investigation to ascertain.

The subgroup analysis of age group showed that calcium levels seemed to be differentially influenced by age, with children showing a significant pooled effect compared to adults. The influence of age on calcium levels was observed in children who were infected with malaria parasites. The differential impact of malaria on calcium levels between children and adults is not fully understood. Calcium metabolism in children is characterized by rapid bone growth, high rates of bone turnover, and the demands of developing and maintaining healthy skeletal structures [51]. During acute illnesses, such as severe malaria, children may be more susceptible to disturbances in calcium metabolism due to their active growth and higher bone turnover compared to adults. Severe malaria, especially cerebral malaria, can lead to various metabolic disturbances, including hypocalcemia [4,32,40,52]. Inadequate calcium and vitamin D intake in children, particularly in malaria-endemic regions, may contribute to lowered calcium levels during infection [53]. The precise mechanisms and reasons for which children might be more susceptible to hypocalcemia during a malarial infection compared to adults are unknown, and more research is needed to fully elucidate this phenomenon.

The sensitivity of our meta-analysis, as evidenced by the leave-one-out approach, underscores the influence of individual studies on the overall effect estimate. While this is a routine facet of meta-analyses, it also prompts a degree of caution in interpreting results. While the review offered a comprehensive outlook, it is essential to recognize the inherent limitations. The pronounced heterogeneity among the studies may obscure some finer nuances. Furthermore, it is important to recognize that another limitation of this study was the lack of uniformity in the specific methods used to evaluate hypocalcemia in children across the included studies. This variability in diagnostic approaches could introduce some heterogeneity into our findings, emphasizing the need for future research to standardize these methods to enable more robust comparisons. Given the variations in observations among individual studies and the absence of statistical significance in the meta-analysis results, future research may benefit from a more detailed exploration, potentially at a regional or population subgroup level.

This systematic review and meta-analysis shed light on the relationship between calcium levels and malaria, revealing a consistent decline in calcium levels among malaria patients across various regions, with children being particularly susceptible. The findings highlighted the potential for calcium monitoring in clinical management and hint at its use as a prognostic biomarker for disease recovery. Notably, geographical differences suggested region-specific strategies might be required in managing malaria. Given the children’s unique vulnerability to hypocalcemia, specialized interventions might be necessary in pediatric populations. Despite the insights provided, marked heterogeneity among the studies underscores the need for further granular research, especially considering regional and age-related nuances. This work served as a foundational step, emphasizing the importance of exploring malaria’s metabolic and clinical impacts, which can guide future research and public health policies.

## 5. Conclusions

This systematic review and meta-analysis have provided crucial insights into the previously underexplored relationship between calcium levels and malaria. Across multiple geographical regions, a discernible trend was identified: a decrease in calcium levels among malaria-infected individuals, with children exhibiting a pronounced susceptibility. The distinct regional variances, especially between Africa and Asia, hint at the complex interplay of factors, including the prevalence of certain *Plasmodium* species and nutritional aspects. The uniquely heightened vulnerability in children suggests potential developmental and metabolic intricacies that warrant further exploration. Future research should delve deeper into regional, age-based, and severity-associated nuances to better inform clinical management strategies, potential prognostic applications, and public health policies centered around malaria’s metabolic repercussions.

## Figures and Tables

**Table 1:** Summary characteristics of the included studies.

Characteristics	*n* (14 Studies)	%
**Publication year**		
Before 2000	4	28.57
2000–2009	5	37.71
2010–2019	4	28.54
2020–2023	1	7.14
**Study designs**		
Cross-sectional study	8	57.14
Case-control study	4	28.57
Cohort study	1	7.14
Clinical trials	1	7.14
**Study areas**		
**Asia**	**7**	50.0
India	3	21.43
Vietnam	2	14.29
Thailand	1	7.14
China	1	7.14
**Africa**	**7**	50.0
Nigeria	3	21.43
Cameroon	2	14.29
Sudan	1	7.14
Kenya	1	7.14
***Plasmodium* species**		
*P. falciparum*	10	71.43
*P. falciparum*, *P. vivax*, mixed infections	2	14.29
*P. falciparum*, *P. vivax*	1	7.14
Not specified	1	7.14
**Participants**		
Children	5	35.71
Adults	7	50.0
Children and adults	2	14.29
**Symptom**		
Symptomatic	11	78.57
Not specified	3	21.43
**Severity status**		
Severe malaria	3	21.43
Severe and non-severe malaria	6	42.86
Non-severe malaria (uncomplicated, mild, or asymptomatic malaria)	1	7.14
Not specified	4	28.57
**Methods for malaria detection**		
Microscopic method	9	64.29
Microscopic method, RDT	2	14.29
Microscopic method, Quantitative buffy coat	1	7.14
Not specified	2	14.29
**Blood sample for calcium**		
Serum	9	64.29
Plasma	5	35.71

Abbreviations: RDT, rapid diagnostic test.

**Table 2:** Comparison of calcium levels in different groups of malaria.

No.	Authors	Continent (Country)	Number of Participants Enrolled	Age Range	*Plasmodium* spp.	Clinical Malaria	Comparison of Calcium Levels in Different Groups of Malaria
1	Abdelsalam et al., 2016 [20]	Africa (Sudan)	Patients with malaria (*n* = 75), normal healthy controls (*n* = 75)	Adults (≥18 years)	*P. falciparum*	Symptomatic malaria	1. Significantly decreased levels of calcium were observed in patients with malaria when compared to uninfected controls. 2. Calcium levels were decreased with increasing parasitemia.
2	Asaolu et al., 2009 [31]	Africa (Nigeria)	Pregnant women: Patients with malaria (*n* = 195), uninfected controls (*n* = 100)	20–43 years	*P. falciparum*	Not specified	Significantly decreased levels of calcium were observed in patients with malaria when compared to uninfected controls.
3	Ayoola et al., 2005 [32]	Africa (Nigeria)	Patients with malaria (*n* = 30), uninfected controls (*n* = 39):	Patients with malaria: 6–108, uninfected controls: 7–108 months	*P. falciparum*	Symptomatic malaria	Significantly decreased levels of calcium were observed in patients with malaria when compared to uninfected controls.
4	Davis et al., 1991 [34]	Asia (Thailand)	Patients with malaria (*n* = 172)	14–72 years	*P. falciparum*	Symptomatic malaria	1. Significantly increased levels of calcium were observed in patients who died when compared to survivors. 2. No difference in levels of calcium was observed in patients with severe malaria when compared to non-severe malaria.
5	Davis et al., 1993 [33]	Asia (China)	*P. falciparum* (*n* = 10), *P. vivax* (*n* = 8), normal healthy controls (*n* = 10)	*P. falciparum*: 11–40, *P. vivax*: 18–38, normal healthy controls: 20–46 years	*P. falciparum*, *P. vivax*	Symptomatic malaria	1. No difference in levels of calcium was observed in patients with malaria (all species) when compared to uninfected controls. 2. Significantly decreased calcium levels were observed in patients with *P. falciparum* malaria compared to uninfected controls. 3. No difference in levels of calcium was observed in patients with *P. vivax* malaria when compared to uninfected controls.
6	Dondorp et al., 2004 [35]	Asia (Vietnam)	Fatal cases (*n* = 46), survivors (*n* = 222)	Fatal cases: 15–74, survivors: 15–79 years	*P. falciparum*	Symptomatic malaria	No difference in levels of calcium was observed between fatal cases and survivors.
7	Inocent et al., 2007 [36]	Africa (Cameroon)	Patients with malaria (*n* = 54), healthy controls (*n* = 62)	≤6 years	*P. falciparum*	Not specified	Significantly decreased levels of calcium were observed in patients with malaria when compared to uninfected controls.
8	Maitland et al., 2005 [37]	Africa (Kenya)	Kenyan children (*n* = 56): survivors (*n* = 42), fatal cases (*n* = 14)	>2 months	*P. falciparum*	Symptomatic malaria	No difference in levels of calcium was observed between fatal cases and survivors.
9	Mfonke et al., 2010 [38]	Africa (Cameroon)	Children who had come for vaccination or counselling (*n* = 45), Uncomplicated malaria (*n* = 94), malaria anemia (*n* = 73), cerebral malaria (*n* = 45), cerebral malaria/malaria anemia (*n* = 13)	6–168 months	*P. falciparum*	Symptomatic malaria	1. No difference in levels of calcium was observed in patients with malaria (uncomplicated, malaria anemia, cerebral malaria) compared to uninfected controls. 2. No difference in levels of calcium between severe (malaria anemia, cerebral malaria) and non-severe malaria.
10	Mishra et al., 2013 [39]	Asia (India)	Patients with malaria (*n* = 70), uninfected controls (not specified number)	5 to >50 years	*P. falciparum*, *P. vivax*, mixed infections	Symptomatic malaria	Significantly decreased levels of calcium were observed in patients with malaria when compared to uninfected controls.
11	Okon et al., 2022 [21]	Africa (Nigeria)	Patients with malaria (*n* = 50), uninfected controls (*n* = 30)	Children (not specified age range)	*P. falciparum*	Not specified	1. No difference in levels of calcium was observed between malaria and uninfected controls. 2. Significantly decreased levels of calcium were observed in patients with severe malaria when compared to mild malaria. 3. Significantly decreased levels of calcium were observed in patients with moderate malaria when compared to mild malaria. 4. No difference in levels of calcium was observed between moderate and severe malaria.
12	Prabha et al., 1998 [40]	Asia (India)	Patients with malaria (*n* = 60)	Adults (not specified age range)	*P. falciparum*, *P. vivax*, mixed infections	Symptomatic malaria	Mean levels of calcium in patients with complicated malaria (falciparum or mixed infections) were lower than those with uncomplicated malaria (falciparum or mixed infections).
13	St. John et al., 1995 [41]	Asia (Vietnam)	Patients with severe malaria (*n* = 25), uninfected controls (*n* = 10)	18–63 years	*P. falciparum*	Symptomatic malaria	Significantly decreased levels of calcium were observed in patients with malaria when compared to uninfected controls.
14	Tyagi et al., 2017 [22]	Asia (India)	Patients with malaria (*n* = 551), uninfected controls (*n* = 211)	13–82 years	Not specified	Symptomatic malaria	Significantly decreased levels of calcium were observed in patients with malaria when compared to uninfected controls.

**Table 3:** Subgroup analyses of calcium levels in patients with malaria compared with uninfected controls.

Subgroup Analyses	*p* Value	Hedges’ g (95% CI)	*I*^2^ (%)	Number of Studies
**Publication years**				
2020–2023	N/A	−0.30 (–0.76–0.15)	N/A	1
2010–2019	0.17	−1.96 (–4.77–0.85)	99.58	3
2000–2009	0.07	−1.72 (–0.39–0.15)	98.02	3
Before 2000	0.37	1.24 (–1.48–3.97)	95.75	2
**Study design**				
Cross-sectional study	0.21	−1.27 (–3.28–0.73)	99.29	5
Case-control study	0.53	−0.65 (–2.67–1.37)	98.39	4
**Continent**				
Africa	0.02	−1.12 (–2.06–(–0.19))	96.89	6
Asia	0.76	−0.70 (–5.25–3.86)	99.41	3
**Age group**				
Children	0.02	−0.42 (–0.78–(–0.05))	69.45	4
Adults	0.55	−0.66 (–2.86–1.53)	98.34	4
Children and adults	N/A	−4.55 (–4.83–(–4.27))	N/A	1
***Plasmodium* species**				
*P. falciparum*	0.23	−0.62 (–1.62–0.38)	97.36	7
*P. vivax*	N/A	−0.14 (–0.89–0.61)	N/A	1
Not specified	N/A	−4.55 (–4.83–4.27)	N/A	1
**Symptoms**				
Symptomatic	0.48	−0.69 (–2.62–1.24)	99.23	6
Not specified	0.11	−1.60 (–3.54–0.35)	98.26	3
**Methods for *Plasmodium* identification**				
Microscopic method	0.23	−1.09 (–2.87–0.69)	99.05	7
Microscopic method, RDT	0.30	−0.66 (–1.92–0.60)	96.46	2
**Blood samples for calcium measurement**				
Serum	0.24	−0.64 (–1.71–0.43)	97.05	7
Plasma	0.31	−2.28 (–6.72–2.15)	99.77	2

Abbreviations: CI, confidence interval; N/A, not assessed.

## Data Availability

All data relating to the present study are available in this manuscript and supplementary files.

## Supplementary Materials

The following supporting information can be downloaded at: https://www.mdpi.com/article/10.3390/nu15214522/s1, Table S1. Search terms; Table S2. Details of included studies; Table S3. Quality of included studies; Table S4. Meta-regression results.
