# Supplementary material for: Regional and Age-Related Variations in Blood Calcium Levels among Patients with Plasmodium falciparum and P. vivax malaria: A Systematic Review and Meta-Analysis
Source: Nutrients. 2023 Oct 25;15(21):4522. doi: 10.3390/nu15214522 (PMC10650696; doi:10.3390/nu15214522)
Supplement: Supplementary file 1 [file nutrients-15-04522-s001.zip › Table S1. Search terms.pdf]

Regional and age-related variations in blood calcium levels among patients with *Plasmodium falciparum* and *P. vivax* malaria: A systematic review and meta-analysis

Kwuntida Uthaisar Kotepui[1†], Aongart Mahittikorn[2†], Polrat Wilairatana[3*], Frederick Ramirez Masangkay[4], Manas Kotepui[1]*

[1]Medical Technology, School of Allied Health Sciences, Walailak University, Thasala, Nakhon Si Thammarat 80160, Thailand

[2]Department of Protozoology, Faculty of Tropical Medicine, Mahidol University, Bangkok 10400, Thailand

[3]Department of Clinical Tropical Medicine, Faculty of Tropical Medicine, Mahidol University, Bangkok 10400, Thailand

[4]Department of Medical Technology, Faculty of Pharmacy, University of Santo Tomas, Manila 1000, Philippines

*Corresponding author

† These authors contributed equally to this work.

Kwuntida Uthaisar Kotepui: kwuntida.ut@wu.ac.th

Aongart Mahittikorn: aongart.mah@mahidol.ac.th

Frederick Ramirez Masangkay: frederick_masangkay2002@yahoo.com

Polrat Wilairatana: polrat.wil@mahidol.ac.th

Manas Kotepui manas.ko@wu.ac.th, Tel+ :.66954392469

General keywords

calcium AND (malaria OR plasmodium OR “Plasmodium Infection“ OR “Remittent Fever“ OR “Marsh Fever“ OR Paludism)

PubMed 23 September 2023

No.	Key concept	Search terms	Results
1.	Calcium	calcium[Text Word] OR calcium[MeSH Terms]	653,505
2.	Malaria	malaria[Text Word] OR malaria[MeSH Terms] OR plasmodium[Text Word] OR "Plasmodium Infection"[Text Word] OR "Remittent Fever"[Text Word] OR "Marsh Fever"[Text Word] OR Paludism[Text Word]	119,506
3.	1 AND 2	(calcium[Text Word] OR calcium[MeSH Terms]) AND (malaria[Text Word] OR malaria[MeSH Terms] OR plasmodium[Text Word] OR "Plasmodium Infection"[Text Word] OR "Remittent Fever"[Text Word] OR "Marsh Fever"[Text Word] OR Paludism[Text Word])	937

Embase 23 September 2023

No.	Key concept	Search terms	Results
1.	Calcium	calcium:ti,ab,kw,de OR calcium/exp	954,039
2.	Malaria	malaria:ti,ab,kw,de OR plasmodium:ti,ab,kw,de OR 'Remittent Fever':ti,ab,kw,de OR 'Marsh Fever':ti,ab,kw,de OR Paludism:ti,ab,kw,de OR malaria/exp	157,922
3.	1 AND 2	(calcium:ti,ab,kw,de OR calcium/exp) AND (malaria:ti,ab,kw,de OR plasmodium:ti,ab,kw,de OR 'Remittent Fever':ti,ab,kw,de OR 'Marsh Fever':ti,ab,kw,de OR Paludism:ti,ab,kw,de OR malaria/exp)	1,622

Scopus 23 September 2023

No.	Key concept	Search terms	Results
1.	Calcium	TITLE-ABS-KEY (calcium)	1,199,200
2.	Malaria	TITLE-ABS-KEY (malaria OR plasmodium OR "plasmodium infection" OR "remittent fever" OR "marsh fever" OR paludism)	158,810
3.	1 AND 2	(TITLE-ABS-KEY (calcium)) AND (TITLE-ABS-KEY (malaria OR plasmodium OR "plasmodium infection" OR "remittent fever" OR "marsh fever" OR paludism))	1,695

Ovid 23 September 2023

No.	Key concept	Search terms	Results

1.	Calcium AND Malaria	calcium AND (malaria OR plasmodium OR "Plasmodium Infection" OR "Remittent Fever" OR "Marsh Fever" OR Paludism)<br><br>Filter: limit to ovid full text available and articles with abstracts and original articles	320
