# Supplementary material for: Regional and Age-Related Variations in Blood Calcium Levels among Patients with Plasmodium falciparum and P. vivax malaria: A Systematic Review and Meta-Analysis
Source: Nutrients. 2023 Oct 25;15(21):4522. doi: 10.3390/nu15214522 (PMC10650696; doi:10.3390/nu15214522)
Supplement: Supplementary file 1 [file nutrients-15-04522-s001.zip › Table S4. Meta-regression results.pdf]

Regional and age-related variations in blood calcium levels among patients with *Plasmodium falciparum* and *P. vivax* malaria: A systematic review and meta-analysis

Kwuntida Uthaisar Kotepui[1†], Aongart Mahittikorn[2†], Polrat Wilairatana[3*], Frederick Ramirez Masangkay[4], Manas Kotepui[1*]

[1]Medical Technology, School of Allied Health Sciences, Walailak University, Thasala, Nakhon Si Thammarat 80160, Thailand

[2]Department of Protozoology, Faculty of Tropical Medicine, Mahidol University, Bangkok 10400, Thailand

[3]Department of Clinical Tropical Medicine, Faculty of Tropical Medicine, Mahidol University, Bangkok 10400, Thailand

[4]Department of Medical Technology, Faculty of Pharmacy, University of Santo Tomas, Manila 1000, Philippines

*Corresponding author

† These authors contributed equally to this work.

Kwuntida Uthaisar Kotepui: kwuntida.ut@wu.ac.th

Aongart Mahittikorn: aongart.mah@mahidol.ac.th

Frederick Ramirez Masangkay: frederick_masangkay2002@yahoo.com

Polrat Wilairatana: polrat.wil@mahidol.ac.th

Manas Kotepui manas.ko@wu.ac.th, Tel+ :.66954392469

Table S4. Meta-regression analysis of covariates on the difference in calcium levels between patients with malaria and uninfected controls

**Covariates**	***P* value**	**tau^2**	***I*^{2} (%)**	**R-squared (%)**	**Number of studies**
Publication years	0.4024	4.949	99.17	0.00	9
Study design	0.6775	4.882	99.06	0.00	9
Continent	0.7718	3.522	98.60	18.95	9
Age group	0.0162	1.702	96.86	60.83	9
Plasmodium species	0.0187	1.772	97.36	59.22	9
Clinical status	0.5699	5.025	99.09	0.00	9
Diagnostic method for malaria	0.8014	4.55	98.94	0.00	9
Types of blood samples	0.3305	4.493	98.91	0.00	9
